# First person – Will Scott

**DOI:** 10.1242/bio.062161

**Published:** 2025-07-31

## Abstract

First Person is a series of interviews with the first authors of a selection of papers published in Biology Open, helping researchers promote themselves alongside their papers. Will Scott is first author on ‘ Fluorescent protein tags for human tropomyosin isoform comparison’, published in BiO. Will is a postdoctoral fellow in the lab of Professor Mohan Balasubramanian at Warwick Medical School, University of Warwick, interested in engineering proteins and synthesising organic molecules to tackle actin cytoskeleton-associated challenges.


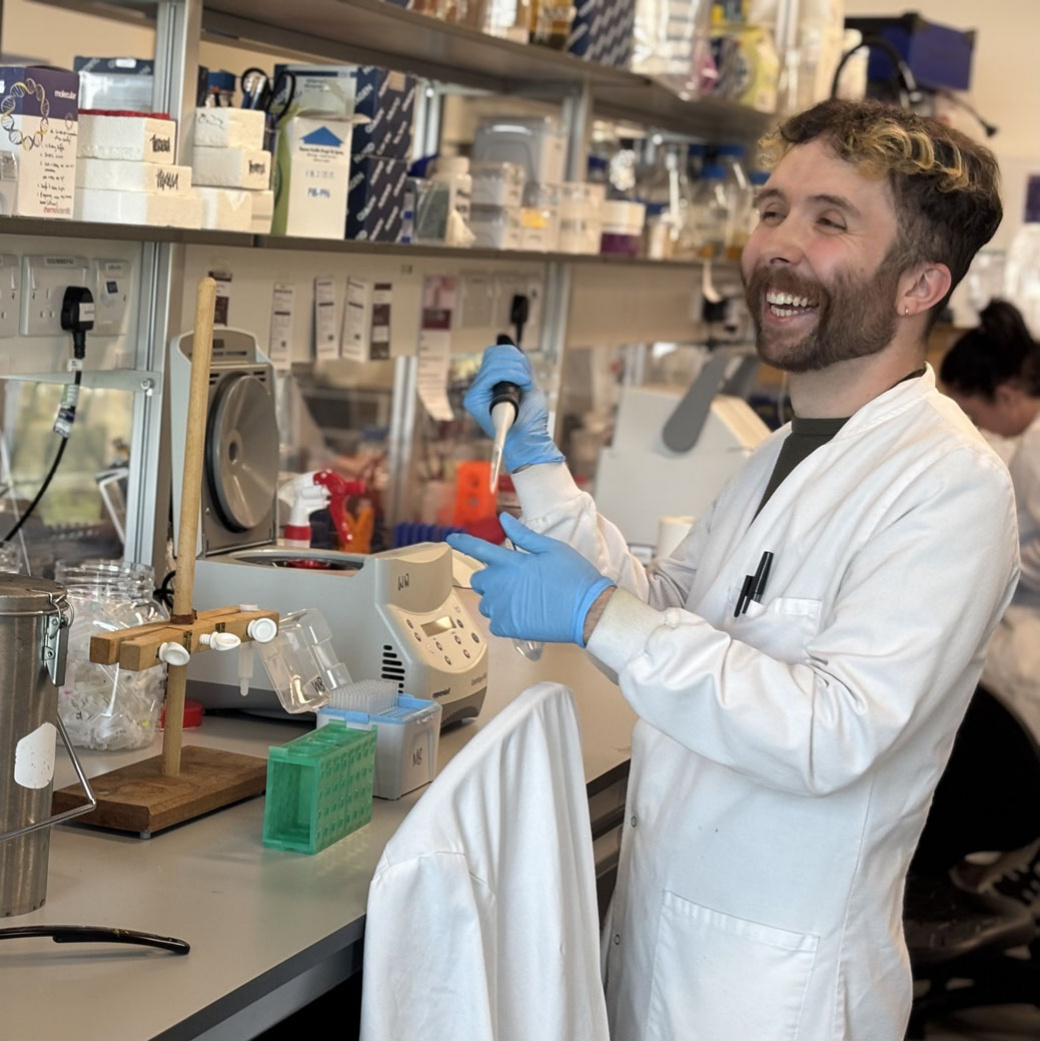


**Will Scott**

**Describe your scientific journey and your current research focus**

I had my first research experiences during undergraduate summers at King's College London. It's a great translational environment. I realised there how fun research is and how impactful it can be. I moved to Warwick for my PhD and really landed on my feet with my supervisor Mohan Balasubramanian, who is a brilliant mentor and has given me the opportunity to flourish.

I am a synthetic biologist primarily, but trained in chemical synthesis during my PhD. I love engineering proteins – you can be so creative with it. I've recently been working on new fluorescent proteins. We published mStayGold(E138D) and StayRose, which have improved photostability, and there's a few more we've designed and are excited to share at some point. My interest is in the actin cytoskeleton, and applying synthetic biology and chemical synthesis principles to tackle cytoskeleton-associated challenges.

**Who or what inspired you to become a scientist?**

The ‘Horrible Science’ book series by Nick Arnold. My Uncle gave me a set of them for Christmas when I was 5 or 6. I was obsessed – I thought I could discover the secret to time travel by reading them all. I didn't discover time travel, but I did discover that I wanted to be a scientist. I made a science club at 9, I was the President (and Head of Genetics). The only other member was my 4-year-old brother, who was Head of Paleontology (he liked dinosaurs).

**How would you explain the main finding of your paper?**

Tropomyosin is a protein in the actin cytoskeleton, a protein network that controls cell shape, movement and division. Tropomyosins form long chains that make it difficult to add fluorescent tags onto them, which is the normal method scientists use to see proteins under a microscope. Our paper employs a strategy for overcoming this problem; by adding a long and flexible linker, we can visualise many different tropomyosins in live human cells with reduced disruption.

by adding a long and flexible linker, we can visualise many different tropomyosins in live human cells with reduced disruption

**What are the potential implications of this finding for your field of research?**

This is a new set of improved tools for use by those wanting to study any of nine human tropomyosins in live (or fixed) cells. We have generated constructs with four different fluorescent proteins – half optimised for still images and the other half for timelapse imaging, all available in both red and green, so plenty of choice. They can be co-expressed with each other to visualise different tropomyosins at the same time.

**Figure:**
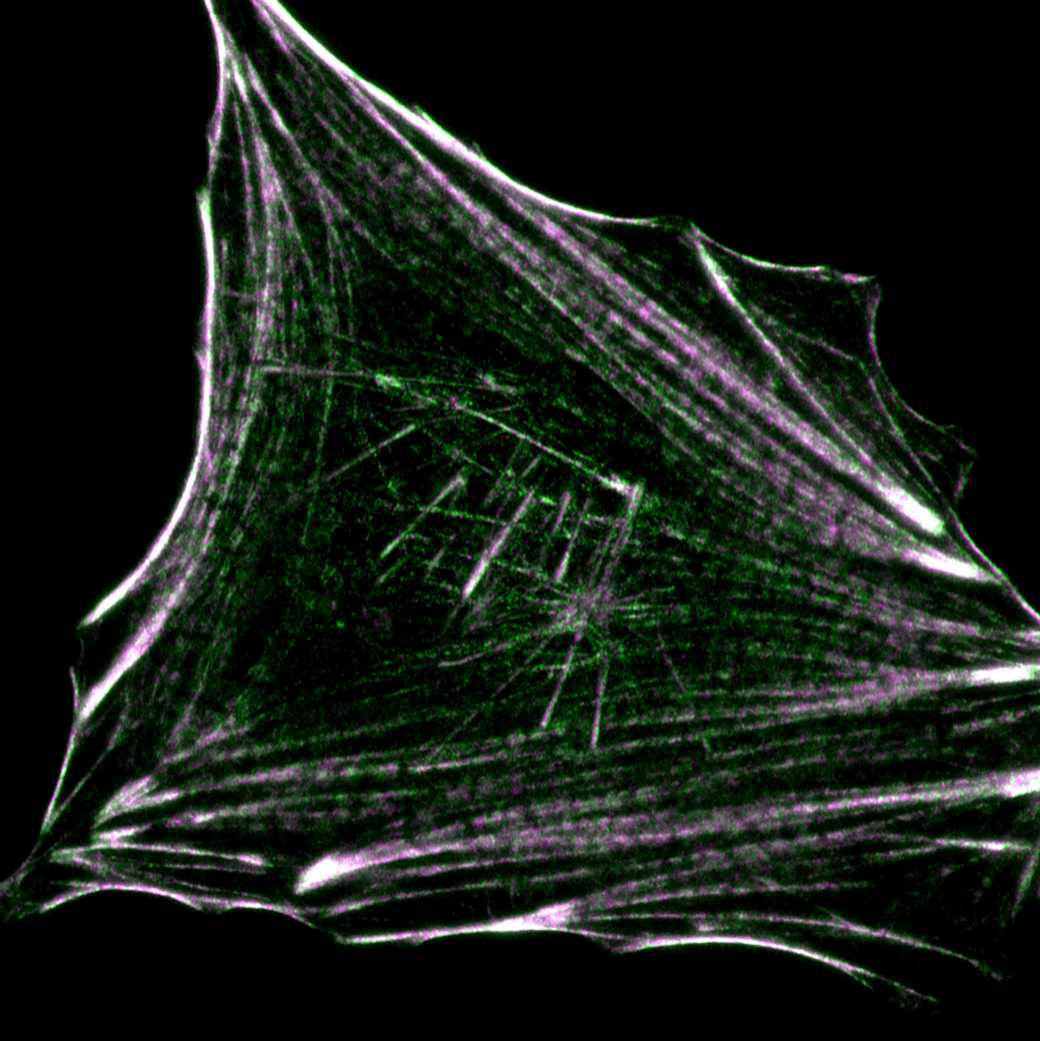
A human retinal cell with two fluorescently tagged tropomyosins.

**Which part of this research project was the most rewarding?**

This project was very unexpected. It started as a favour to a colleague during the second year of my PhD. She needed a mammalian version of her yeast tropomyosin construct to finish up her paper, which we published in 2022 in Journal of Cell Science. Thirty-five extra constructs later, it evolved into a chapter of my thesis and now into another publication. I didn't foresee this at all, and what a pleasant surprise.

**What do you enjoy most about being an early-career researcher?**

The freedom to pursue your interests is what drew me to this career path. It's a job that you can design and redesign as your interests evolve. Not many jobs offer that. It feels like a hobby that you get paid for. I love the people I work with and we have such a laugh. Getting to do research with them is very fulfilling and the prospect of making an impact is the icing on the cake.

**What piece of advice would you give to the next generation of researchers?**

The advice I remind myself of is to use your creativity and be intentional. Research is as creative as any artform. Applying a creative mindset allows you to overcome problems in a way only you know how to, as well as take ownership of your research. In terms of being intentional, choose projects that will train you in many widely applicable skills, that way you still have the skills, whatever the outcome of the research is. Choose a supervisor who genuinely wants to nurture your career, which can be hard to screen for before joining a lab, so talk to current and previous lab members.

Research is as creative as any artform.

**What's next for you?**

I'm aiming to secure funding to apply my skills in synthetic biology, organic chemistry and the actin cytoskeleton to the issue of aberrant endothelial wound healing, which underpins many autoimmune diseases. As for the immediate future, I'm working on a few projects that are nearing completion. I'm particularly excited about one that features some novel protein dyes I synthesised and employs a genetic code expansion approach.

## References

[BIO062161C1] Scott, W., Polutranko, V., Milczarek, J., Hands-Portman, I. and Balasubramanian, M. K. (2025) Fluorescent protein tags for human tropomyosin isoform comparison. *Biol. Open.* 14, bio061992. 10.1242/bio.061992 40635464 PMC12352278

